# *Lvrn* expression is not critical for mouse placentation

**DOI:** 10.1262/jrd.2018-157

**Published:** 2019-02-10

**Authors:** Tomohiro TOBITA, Daiji KIYOZUMI, Masanaga MUTO, Taichi NODA, Masahito IKAWA

**Affiliations:** 1) Research Institute for Microbial Diseases, Osaka University, Osaka 565-0871, Japan; 2) Graduate School of Medicine, Osaka University, Osaka 565-0871, Japan; 3) Immunology Frontier Research Center, Osaka University, Osaka 565-0871, Japan; 4) The Institute of Medical Science, The University of Tokyo, Tokyo 108-8639, Japan

**Keywords:** Knockout, Lentivirus, Trophoblast

## Abstract

Preeclampsia is a systemic disease caused by abnormal placentation that affects both mother and fetus. It was reported that *Laeverin* (*LVRN*, also known as Aminopeptidase Q) was up-regulated in the placenta of preeclamptic patients. However, physiological and pathological functions of *LVRN* remained to be unknown. Here we characterized *Lvrn* function during placentation in mice. RT-PCR showed that *Lvrn* is expressed in both fetus and placenta during embryogenesis, and several adult tissues. When we overexpressed *Lvrn* in a placenta-specific manner using lentiviral vectors, we did not see any defects in both placentae and fetuses. The mice carrying *Lvrn* overexpressing placentas did not show any preeclampsia-like symptoms such as maternal high blood pressure and fetal growth restriction. We next ablated *Lvrn* by CRISPR/Cas9-mediated genome editing to see physiological function. In *Lvrn* ablated mice, maternal blood pressure during pregnancy was not affected, and both placentas and fetuses grew normally. Collectively, these results suggest that, LVRN is irrelevant to preeclampsia and dispensable for normal placentation and embryonic development in mice.

The placenta is an essential organ for fetal development through nutrient transport, gas exchange, and hormone secretion. Abnormalities in placental formation and function trigger pregnancy disorders such as preeclampsia (PE) and threatens both maternal and fetal lives [1,2,3,4]. PE occurs in 3–5% of all pregnant women and is characterized by new onset of maternal high blood pressure after 20 weeks of gestation together with other systemic symptoms such as renal dysfunction, or retarded fetal growth [5, 6]. Insufficient trophoblast cell invasion, accompanying oxidative stress and endothelial dysfunction were regarded as the cause of PE [7, 8].

Global gene expression analysis revealed that numerous genes were dysregulated in the PE placenta (reviewed in [9]). *Laeverin* (*LVRN*, also known as Aminopeptidase Q), which encodes a type-II transmembrane M1 aminopeptidase, is one gene that is upregulated in the extravillous trophoblast from PE placentas (up-regulated 10.0 fold compared to control [10], up-regulated 2.4 fold compared to control [11]). In human, *LVRN* is specifically expressed in extravillous trophoblastt [12]. From *in vitro* experiments using primary human extravillous trophoblast and the BeWo cell line, LVRN appears to be involved in trophoblast invasion [13]. However, the relationship between *LVRN* expression levels and pathogenesis of PE is still unknown.

Here we examined physiological and pathological functions of LVRN by lentiviral vector mediated placenta-specific overexpression [14, 15] and CRISPR/Cas9 mediated gene knockout [16] in mice.

## Materials and Methods

### Animals

All animal experiments were approved by the Institutional Animal Care and Use Committee of the Research Institute for Microbial Diseases, Osaka University (H30-01-0). The *Lvrn* mutant mouse line B6D2-Lvrn<em1Osb> will be available to the scientific community through RIKEN BRC (http://mus.brc.riken.jp/en/).

### RT-PCR

cDNAs were synthesized from various tissues using Trizol and SuperScript IV (Thermo Fisher Scientific, Waltham, MA, USA). cDNAs synthesized from 10 ng of total RNA were used for RT-PCR as templates using KOD Fx neo (TOYOBO, Osaka, Japan) with the following primers: forward; 5’-CGCAATGAGCTGCAGTAAAGACCC-3’ and reverse; 5’-CAGGCACTAGAGCATCCAGCC-3’ for *Lvrn*, forward; 5’- CATCCGTAAAGACCTCTATGCCAAC-3’ and reverse; 5’-ATGGAGCCACCGATCCACA-3’ for *Actb*. The amplification cycles were 94 degrees for 30 sec, 65 degrees for 30 sec, and 72 degrees for 30 sec for 40 cycles. The expected amplicon sizes for *Lvrn* and *Actb* are 216 bp and 171 bp, respectively.

### Antibodies

A polyclonal antibody against mouse LVRN (NM_083284) was raised in rabbit by immunizing with the synthetic peptide CKNLQNKKRIARVVEWLRKNT (amino acids 972–991) conjugated with keyhole limpet hemocyanin. Antiserum was purified by affinity chromatography with Sulfolink coupling gel (Thermo Fisher Scientific) conjugated with antigenic peptide. A rabbit monoclonal antibody against mouse GAPDH (14C10) was purchased from CST (Cell Signaling Technology, Danver, MA, USA). A mouse monoclonal antibody against beta-actin (AC-15) was from Abcam (Cambridge, UK). A rat monoclonal antibody against EGFP (K2) was generously gifted from S.C. Fujita at Mitsubishi Institute of Life Sciences, Tokyo, Japan. A rat monoclonal antibody TROMA-1 (MABT239) was purchased from Merck Millipore (Darmstadt, Germany). Alexa Fluor 546-conjugated goat anti-rabbit IgG antibody (A11006) and Alexa Fluor 488-conjugated goat anti-rat IgG antibody (A11071) were purchased from Thermo Fisher Scientific. A goat polyclonal antibody against rabbit IgG conjugated with horseradish peroxidase (111-035-003) and a goat polyclonal antibody against mouse IgG conjugated with horseradish peroxidase (115-035-003) were both from Jackson Immunoresearch (West Grove, PA, USA).

### Preparation of lentiviral vectors

The HIV-1-based, self-inactivating lentiviral vectors were prepared as described previously [14]. Mouse *Lvrn* cDNA was amplified from E18.5 placental cDNAs with the following primers: forward; 5’-CCCCGCTAGCGCCGCCATGAGCCGTCCTTTCAGCTCC-3’ and reverse; 5’-CCCCGTCGACTTACGTGTTTTTCCGAAGCCACTC-3’. A 3.0 kb *Lvrn* fragment was cloned into pLV-CAG 1.2 using NheI and SalI sites to generate pLV-*Lvrn*. Lentiviral vectors are prepared as described previously [14]. In brief, the pLV-*Egfp* and pLV-*Lvrn* plasmids were transfected to 293T cells together with pMDL, pRev, and pVSV-G by the calcium phosphate method. Lentiviral vectors were harvested 2 days after transfection, and concentrated 1,000-fold by ultracentrifugation (first centrifuge; 19,400 rpm, 120 min, second centrifuge; 21,000 rpm, 90 min). After resuspension of precipitates with Hanks Balanced Salt Solution buffer, the concentration of LV-*Lvrn* was determined by measuring p24 gag antigens with an Enzyme-Linked Immunosorbent assay (ELISA) kit (Zeptometrix, Buffalo, NY, USA).

### Lentiviral transduction of mouse blastocysts

Blastocysts collected from B6D2F1 females (SLC) were treated with acidic Tyrode solution (Sigma-Aldrich, St. Louis, MO, USA) to remove the zona pellucida. The zona pellucida-free blastocysts were incubated for 5 hours with LV-*Lvrn* or LV*-Egfp* lentiviral vectors at a concentration of 2.0 × 10^3^ or 8.0 × 10^3^ ng/ml of p24 diluted in KSOM medium. The transduced blastocysts were implanted into the uteri of pseudopregnant E2.5 ICR female mice. Fifteen blastocysts were implanted into each horn of the uterus. Placenta-specific gene transduction was confirmed by genomic PCR with the following primer pairs: forward; 5’- GGGAAGTTATTTATGATGTG-3’ for LV-*Lvrn*, forward; 5’- ACCATGGTGAGCAAGGGCGAG-3’ for LV-*Egfp* with common WPRE primer: reverse; 5’- GGCATTAAAGCAGCGTATCCAC-3’.

### Generation of Lvrn mutant mice by CRISPR/Cas9

The pX330-*Lvrn* plasmid expresses hCas9 and sgRNA targeting mouse *Lvrn* were prepared by ligating annealed oligonucleotides (forward; 5’-CACCGCGTCTATGTGAGCCGCGGG-3’ and reverse; 5’-AAACCCCGCGGCTCACATAGACGC-3’) into the BbsI site of pX330 (http://www.addgene.org/ 42230/). The resulting pX330-*Lvrn* plasmid was injected into one pronucleus of B6D2F1 × B6D2F1 fertilized eggs as previously described [16]. Injected eggs were cultured in KSOM medium overnight and transferred into oviducts of pseudopregnant ICR females. The resulting pups were genotyped by genomic PCR with the primers: forward; 5’-AGTCTTCTCGGGCTCCTAGAGGAG-3’ and reverse; 5’-GTGAGCGCAGCTGCCATACAAGG-3’ and direct DNA sequencing.

### Measurement of blood pressure

Blood pressure (BP) was measured by the tail-cuff method with BP98A (Softron, Tokyo, Japan) as described previously [22]. The pregnant mice were gently secured in a small net without anesthesia. After their behavior, heart rates, and blood pressures were stabilized, both systolic blood pressure (SBP) and diastolic blood pressure (DBP) were measured at least five times. The blood pressure of each embryonic day were also measured in at least five individual females. SBP and DBP data from pregnant mice carrying three to fourteen pups were used for further statistical analysis.

### Immunoblotting

Tissues were homogenized with lysis buffer (20 mM Tris-HCl pH 7.4, 150 mM NaCl, 1% Triton X-100) containing 1% protease inhibitor cocktail (Nacalai Tesque, Kyoto, Japan). Homogenates were centrifuged at 12,000 rpm for 15 min at 4 degrees. The resulting supernatants were recovered and their protein concentration was quantified via the Bradford method. Twenty μg of protein were separated by SDS-PAGE under reducing conditions and transferred onto PVDF membranes using Transblot Turbo (Bio-Rad, Munich, Germany). After blocking with 3% of BSA in TBST, membranes were incubated with primary antibodies (anti-LVRN, 3 μg/ml; anti-b-actin, 1/1000; anti-GAPDH, 1/1000) diluted in 3% BSA in TBST overnight at 4 degrees, After washing with TBST, membranes were incubated with secondary antibodies conjugated with horseradish-peroxidase at room temperature for 30 min (1/10,000 dilution). After washing with TBST, the bound primary antibodies were visualized with using Chemi-Lumi One Super (Nacalai Tesque).

### Histology

Female pregnant mice were sacrificed on the following days of pregnancy 14.5, and 18.5. Embryos and placentas were collected and weighed. Placentas were fixed in 4% paraformaldehyde (Wako, Osaka, Japan) in PBS, embedded in paraffin, and sectioned at 8 μm thickness. Specimens were stained with Mayer hematoxylin solution (Wako) and Eosin solution (Wako). Specimens were mounted in Permount (Falma, Tokyo, Japan) and analyzed with a BZ-X710 microscope (Keyence, Osaka, Japan).

### Immunofluorescence

Rehydrated paraffin sections were incubated with blocking solution (2% goat serum in PBS) at room temperature for 1 h. Specimens were incubated with 3.0 μg/ml of rabbit anti-LVRN antibody diluted in blocking solution at 4 degrees at overnight. After washing with PBS, specimens were incubated with Alexa Fluor 546-conjugated goat anti-rabbit IgG antibody (1/400 diluted) and 0.1 μg/ml of Hoechst 33342 in blocking solution at room temperature for 1.5 h. After washing with PBS, specimens were sealed with 10% glycerol in PBS and analyzed with a BZ-X710 microscope.

### Statistical analysis

All data are shown as the mean ± SD of at least three independent experiments. Statistical analyses were performed using Student’s *t*-test after the data were tested for normality of distribution. Values under 0.05 were regarded as significant.

## Results

### Expression of Lvrn in mouse tissues

**Fig. 1.**
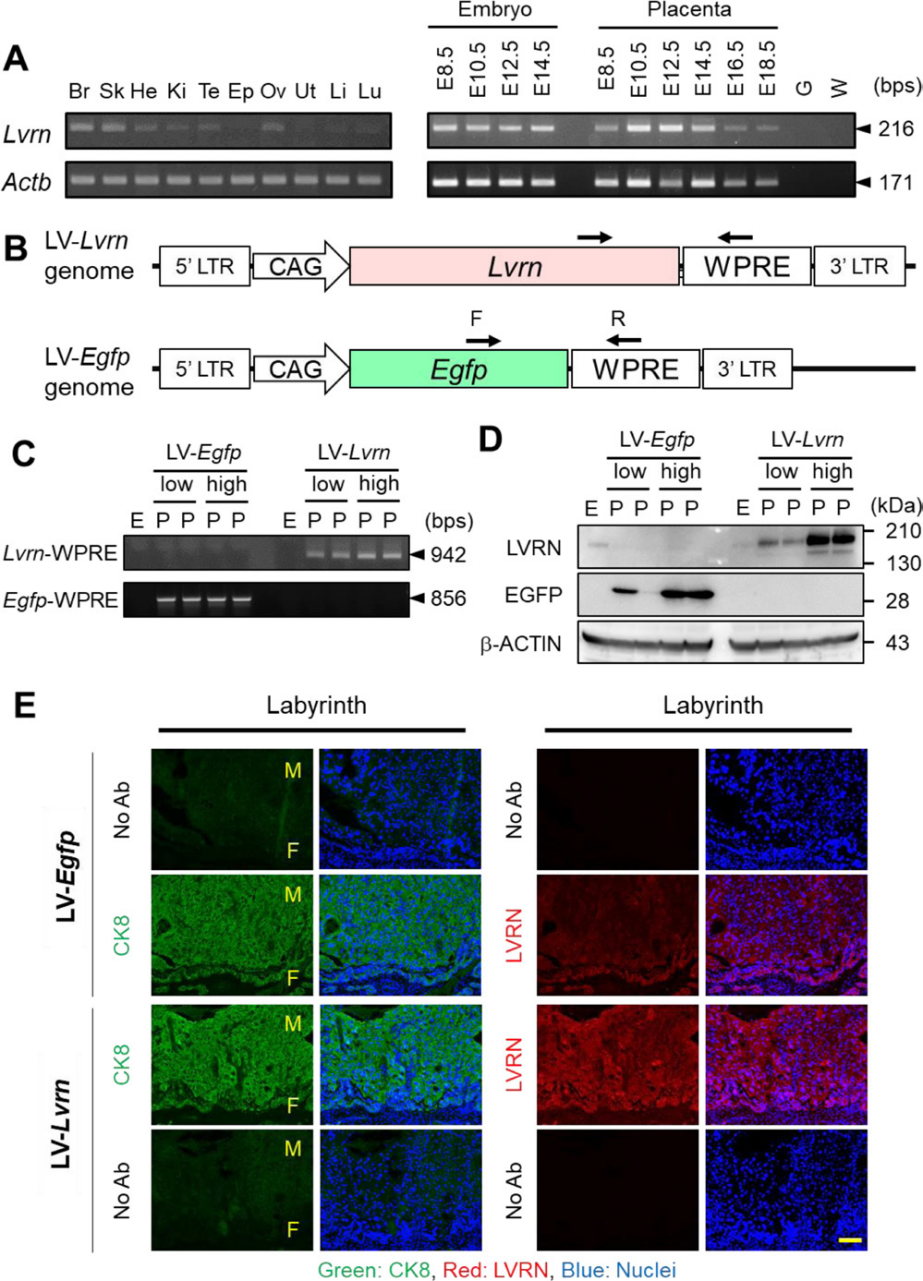
Generation of lentiviral vector-mediated placenta-specific *Lvrn* overexpression mice. (A) RT-PCR analysis of *Lvrn* expression. Left, adult tissues. Br: Brain, Sk: Skin, He: Heart, Ki: Kidney, Te: Testis, Ep: Epididymis, Ov: Ovary, Ut: Uterus, Li: Liver, Lu: Lung. Right, *Lvrn* mRNA expression in whole embryonic and placental tissues at different embryonic stages (40 cycles). *Actb* expression was used as a positive control (28 cycles). E: Embryonic day, G: Genomic DNA, W: Water. (B) Schematic representation of LV-*Lvrn* and LV-*Egfp* constructs. CAG: CAG promoter, WPRE: Woodchuck hepatitis virus posttranscriptional regulatory element, F: Forward primer, R: Reverse primer, LTR: long terminal repeat. (C) Detection of the integrated lentiviral genome by genomic PCR. Integration of LV-*Lvrn* or LV-*Egfp* was detected with specific primers for each lentivirus. (D) Immunoblot detection of LVRN and EGFP protein expression in LV-transduced embryos and placentas. Concentration of lentivirus used are 2.0 × 10^3^ ng/ml (low) or 8.0 × 10^3^ ng/ml (high). (E) Immunofluorescence staining of CK8 (Right) and LVRN (Left) in LV-*Egfp* or LV-*Lvrn* transduced placentas, Green, CK8; Red, LVRN; Blue, Nuclei. F; fetal side, M; Maternal side, Scale Bar; 100 μm.

We performed RT-PCR and found that *Lvrn* mRNA is expressed ubiquitously in adult mouse organs such as brain, skin, heart, kidney, testis and ovary (Fig. 1A

). During gestation, *Lvrn* mRNA was detected in both fetus and placenta at all examined embryonic ages, (E) 8.5, 10.5, 12.5, 14.5, 16.5, and 18.5 (Fig. 1A). Ubiquitous expression in adult and continuous expression during embryonic development suggests that LVRN might play fundamental functions *in vivo*.

### Placenta-specific Lvrn overexpression in mice

To examine the effects of *Lvrn* overexpression on placental formation and function, we utilized lentiviral vector (LV)-mediated placenta-specific gene expression. We prepared third generation lentiviral vectors expressing control EGFP or murine *Lvrn* under the strong ubiquitous CAG promoter [14] (Fig. 1B). We transduced blastocyst stage embryos at two different concentrations, 2.0 × 10^3^ p24-ng/ml and 8.0 × 10^3^ p24-ng/ml, after removal of the zona pellucida. The transduced blastocysts were transplanted into the uteri of pseudopregnant female mice.

Placenta-specific viral vector integration was examined by genomic PCR. The amplicons were only detected in placentae but not in fetuses from LV-transduced embryos (Fig. 1C). When we performed immunoblot analysis, EGFP protein was detected only in LV-Egfp transduced placentas (Fig. 1D). For LVRN, we detected very weak endogenous signals in fetuses and placentae from non-transduced samples (Fig. 1D). After LV-*Lvrn* transduction, we detected strong signals in the placentas dose-dependently, suggesting LVRN is overexpressed in these placentas (Fig, 1D). Trophoblast-specific overexpression of LVRN protein in placental tissue was also confirmed by immunohistochemistry with antibodies against LVRN and CK8 (trophoblast marker, Cytokeratin 8) (Fig. 1E). There were no obvious differences in trophoblast giant cell invasion.

### Pathological functions of LVRN in mouse placenta

**Fig. 2.**
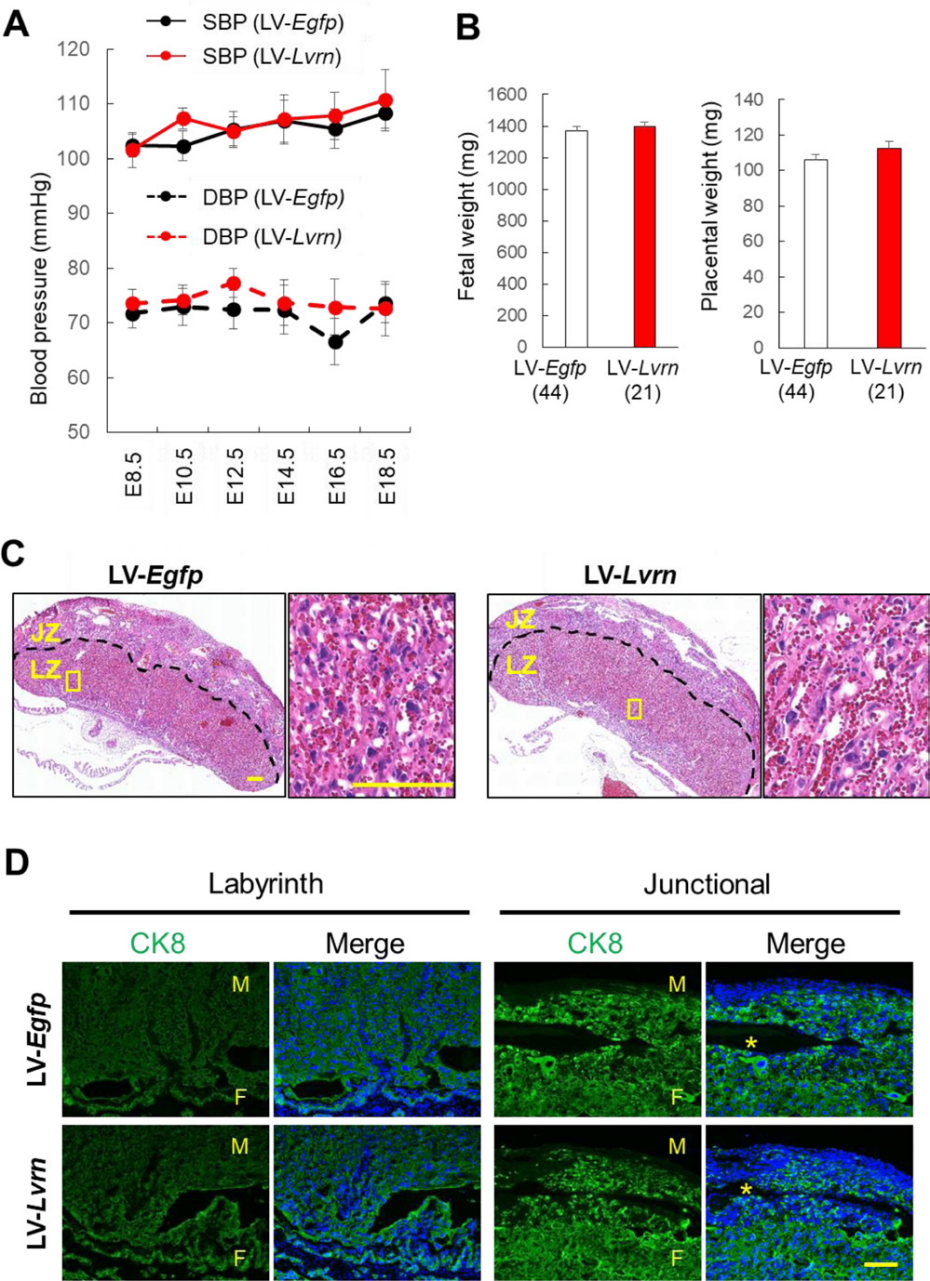
Analysis of placenta-specific *Lvrn* overexpression mice. (A) Blood pressures of recipients with embryos transduced with 8.0 × 10^3^ ng/ml of LV-*Egfp* or LV-*Lvrn*. Systolic and diastolic blood pressures are indicated with solid and dotted lines, respectively. Each point and bar represent average and standard deviation, respectively. (B) Fetal and placental weights of LV-*Egfp* or LV-*Lvrn* (8.0 × 10^3^ ng/ml) transduced embryos recovered at E18.5. Each bar represents the average and standard deviation. (C) Left, hematoxylin-eosin staining of LV-transduced placental sections. Dotted line demarcates the labyrinth zone (LZ) and junctional zone (JZ). Right, magnified images of boxed area in left. Scale Bars, 300 μm (Left), 100 μm (Right). (D) Immunofluorescent staining of CK8 in LV-*Egfp* or LV-*Lvrn* transduced placentas. Asterisk, Maternal blood sinus. F, fetal side, M, Maternal side, Scale Bar, 100 μm.

To assess the effects of placenta-specific overexpression of LVRN, we observed maternal blood pressure, fetal development, and placental histology. We obtained comparable numbers of healthy fetuses and placentas by Caesarian section at E18.5. No significant differences in the blood pressures were observed throughout gestation (Fig. 2A

) and no significant differences in fetal and placental weight (Supplementary Table 1: online only, Fig. 2B). From HE staining, we could not detect any overt abnormalities in the placentas overexpressing LVRN (Fig. 2C). Immunohistochemical staining of CK8 revealed that there were no obvious differences in trophoblast giant cell invasion (Fig. 2D). These results suggest that placenta-specific *Lvrn* overexpression does not interfere with pregnancy in mice.

### Generation of Lvrn-KO mice

**Fig. 3.**
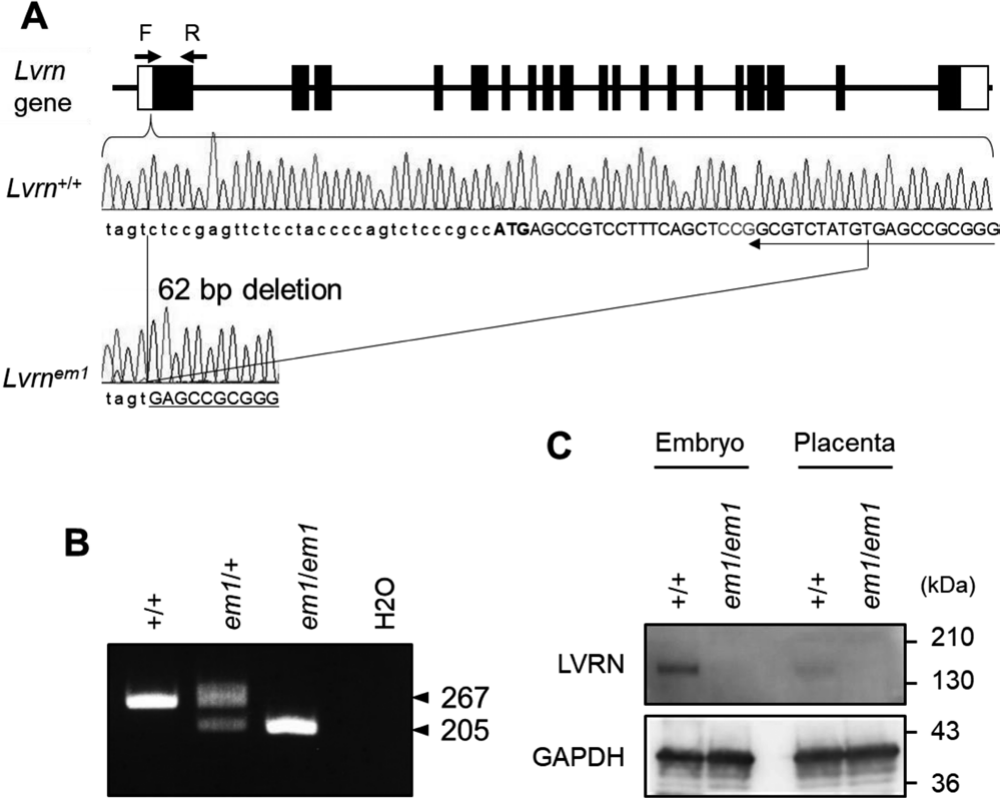
Generation of *Lvrn*-KO mice by CRISPR/Cas9-mediated genome editing. (A) Schematic diagram for generating the *Lvrn* mutant allele. Gene structure of mouse *Lvrn*. Boxes indicate exons. Filled area indicates protein coding regions. Both WT and *Lvrn^em1^* genomic sequences around the initial codon are shown. Bold, Initial ATG codon; Red, Proto-spacer Adjacent Motif sequence; Arrow, single-guide RNA sequence. In the *Lvrn^em1^* mutant allele, a 62 bp region including the initial codon is missing. (B) *Lvrn* genotyping by genomic PCR. WT *Lvrn* and *Lvrn^em1^* alleles give 267 bp and 205 bp amplicons, respectively. (C) LVRN protein expression in WT and *Lvrn^em1/em1^* mutant mice*.* Embryonic and placental proteins were immunoblotted with anti-LVRN antibody. GAPDH was also detected as a positive control.

To further elucidate LVRN function *in vivo*, we next generated *Lvrn*-KO mice using CRISPR/Cas9. We designed single-guide RNA targeting 20 nts immediately downstream of the translational initiation site (Fig. 3A

). There were no off-target sites identified that matched 12 nts at the 3’ end plus the PAM sequence. We injected 129 B6D2F1 × B6D2F1 zygotes with pX330 plasmid expressing the *Lvrn* targeting sgRNA and Cas9 simultaneously. Among the 14 pups obtained, we found a pup carrying a 62 bp deletion at the targeted site (Supplementary Table 2: online only, Fig. 3A). Germline transmission of the mutation was determined by genomic PCR (Fig. 3B). We did not see any signal in both fetus and placenta with anti-LVRN (C-terminus) immunoblot analysis, indicating the LVRN was ablated in *Lvrn^em1/em1^* mutant mice (Fig. 3C).

### Physiological functions of LVRN in mouse placenta

**Fig. 4.**
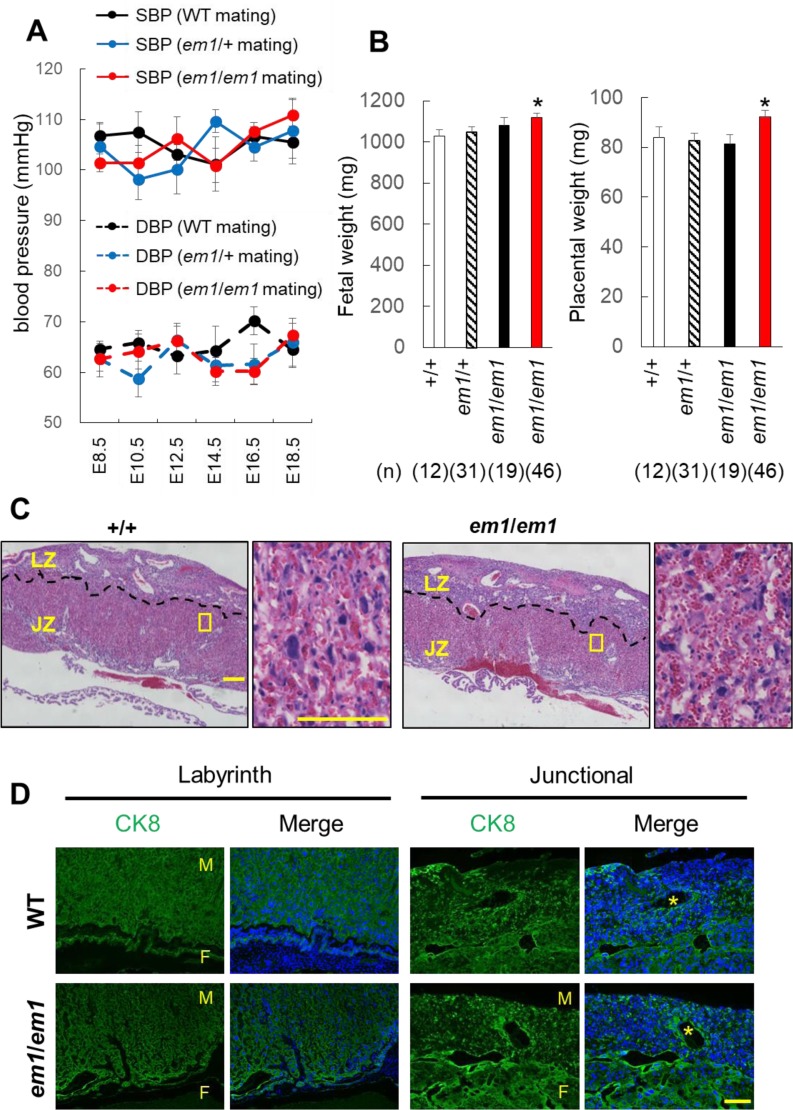
Placental histology and function were not critically affected in the absence of LVRN. (A) Maternal blood pressure during pregnancy of WT, heterozygous, and homozygous matings. Systolic and diastolic blood pressures are indicated with solid and dotted lines, respectively. Each point and bar represent the average and standard deviation, respectively. (B) Fetal (Right) and placental (Left) weight recovered from E18.5 of heterozygous or homozygous matings. WT; wild-type, *: *Lvrn^em1/em1^* pups from *Lvrn^em1/em1^* male and female matings. (C) Left; hematoxylin-eosin staining of WT and homozygous placenta at E18.5. Dotted line demarcates the labyrinth zone (LZ) and junctional zone (JZ). Right; magnified images of boxed area in left. Scale Bars; 300 μm (Left), 100 μm (Right). (D) Immunofluorescent staining of CK8 in WT and *Lvrn^em1/em1^* mutant placentas. Asterisk, Maternal blood sinus, F; fetal side, M; Maternal side, Scale Bar; 100 μm.

To assess the effects of *Lvrn* disruption, we mated *Lvrn*^+/^*^em1^* females with *Lvrn*^+/^*^em1^* males and observed fetal development, maternal blood pressure, and placental histology. We obtained healthy fetuses in Mendelian ratios (+/+ : +/*em1* : *em1*/*em1* = 19 : 40 : 23 from 10 litters, Supplementary Table 3: online only) with no significant differences in maternal blood pressure (Fig. 4A

) throughout gestation, fetal and placental weight among the groups (Fig. 4B). To eliminate the contribution of maternal LVRN, we mated *Lvrn^em1^*^/^*^em1^* females with *Lvrn^em1^*^/^*^em1^* males. From these homozygous crosses, we did not see any defects in fetal and placental development (Fig. 4B). Histological analysis did not show any overt abnormalities (Fig. 4C) and trophoblast invasion into maternal spiral artery in *Lvrn* KO placentas (Fig. 4D). It should be noted that no obvious differences were observed at E14.5 (Supplementary Fig. 1: online only). These results suggest that LVRN is not required for embryonic development, placental formation and function in mice.

## Discussion

Preeclampsia is a systemic disease caused by abnormal placentation and affects both mother and fetus. Epidemiological studies have suggested the biological molecules contributing to placental vasculogenesis and controlling maternal blood pressure as the causes or exacerbating factors in preeclampsia (e.g., soluble Fms-like tyrosine kinase (sFLT1) [17], soluble Endoglin (sENG) [18], transcriptional factors such as STOX1 [19], and protein peptidases (ADAMs, MMPs) [20, 21]). Further, their physiological and pathological roles in preeclampsia have been elucidated using gene manipulated animals [22,23,24,25]. We have previously demonstrated that he LV-mediated placenta specific sFLT1 gene expression resulted in preeclampsia in mice [22]. In the present study, we selected *Lvrn,* a highly expressed gene in preeclamptic placentas [10, 11] and analyzed its role using the same LV-mediated placenta specific expression approach. However, we did not see any preeclamptic symptoms in the treated mice. We further determined the LVRN physiological functions by making CRISPR/Cas9 mediated KO mice. The gene deletion did not cause any defects in pregnancy. Although LVRN overexpression might be used as a biomarker in human, we conclude that the LVRN is dispensable for placentation and placental functions in mice.

In human, LVRN is an active M1 aminopeptidase that degrades Kisspeptin-10 and promotes extravillous trophoblast invasion [26]. The consensus peptide recognition sequence of LVRN aminopeptidase is HXMEN. In rodents, Histidine has been substituted with Glycine and altered LVRN’s substrate specificity [27]. Moreover while human *LVRN* is specifically expressed in extravillous trophoblast [12], *Lvrn* mRNA was ubiquitously detected in mice (Fig. 1A). These differences may explain our negative results and will give us a lead to understand the physiological significances of LVRN in different species. The placenta-specific overexpression of human *LVRN* in mouse should also be tried as we generated preeclamptic model mice with human sFLT1.

Besides, differences in repertoires of aminopeptidases in human and mouse need to be considered. Human and mouse have 13 and 11 aminopeptidases, respectively. It is reported that the disruption of *Enpep* (aminopeptidase A) that degrades Angiotensin-II resulted in elevated blood pressure baseline symptoms in mice [28]. Several SNIPs have been found in ERAP2 (Endoplasmic reticulum aminopeptidase 2) gene in preeclamptic patients. Comparative studies on the different aminopeptidases would also be beneficial to elucidate the unique function of LVRN.

In conclusion, we show *Lvrn*, as a single factor, is irrelevant to preeclampsia and dispensable for normal placentation and embryonic development in mice. By taking advantages of LV vectors, one can introduce multiple genes in a single cell. The CRISPR/Cas9 approach also allows us to delete multiple genes from a single cell. These approaches will shed light on the combined effects of the family genes on placentation and placental functions.

## Supplementary

Supplementary Methods
