# Supplementary material for: Lvrn expression is not critical for mouse placentation
Source: J Reprod Dev. 2019 Feb 10;65(3):239–44. doi: 10.1262/jrd.2018-157 (PMC6584185; doi:10.1262/jrd.2018-157)
Supplement: Supplementary Methods [file jrd-65-239-s001.pdf]

Supplementary Table 1. Developmental ratio of LV-transduced embryos

LV<br>(x p24 10^3 ng/ml)	LV-*Egfp*<br>(2.0)	LV-*Egfp*<br>(8.0)	LV-*Lvrn*<br>(2.0)	LV-*Lvrn*<br>(8.0)
Developmental rate<br>(pups/transfer)	13.9%<br>(20/144)	22.5%<br>(40/178)	43.3%<br>(52/120)	32.4%<br>(67/207)

Supplementary Table 2. Survival rate and indel efficiency of pX330-*Lvrn* embryos

Injected	2-cell	Recipient	Pregnant	Pups	Analyzed	GMO
129	77	5	4	14	12	9

Supplementary Table 3. The genotype of pups obtained from *Lvrn*$^{em1/+}$ intercrosses

	Lvrn$^{+/+}$	*Lvrn*$^{em1/+}$	*Lvrn*$^{em1/em1}$
Litter 1	2	1	4
Litter 2	2	3	3
Litter 3	3	9	0
Litter 4	1	8	1
Litter 5	1	4	2
Litter 6	2	2	2
Litter 7	3	4	1
Litter 8	2	3	2
Litter 9	2	4	4
Litter 10	1	2	4
Total	19	40	23

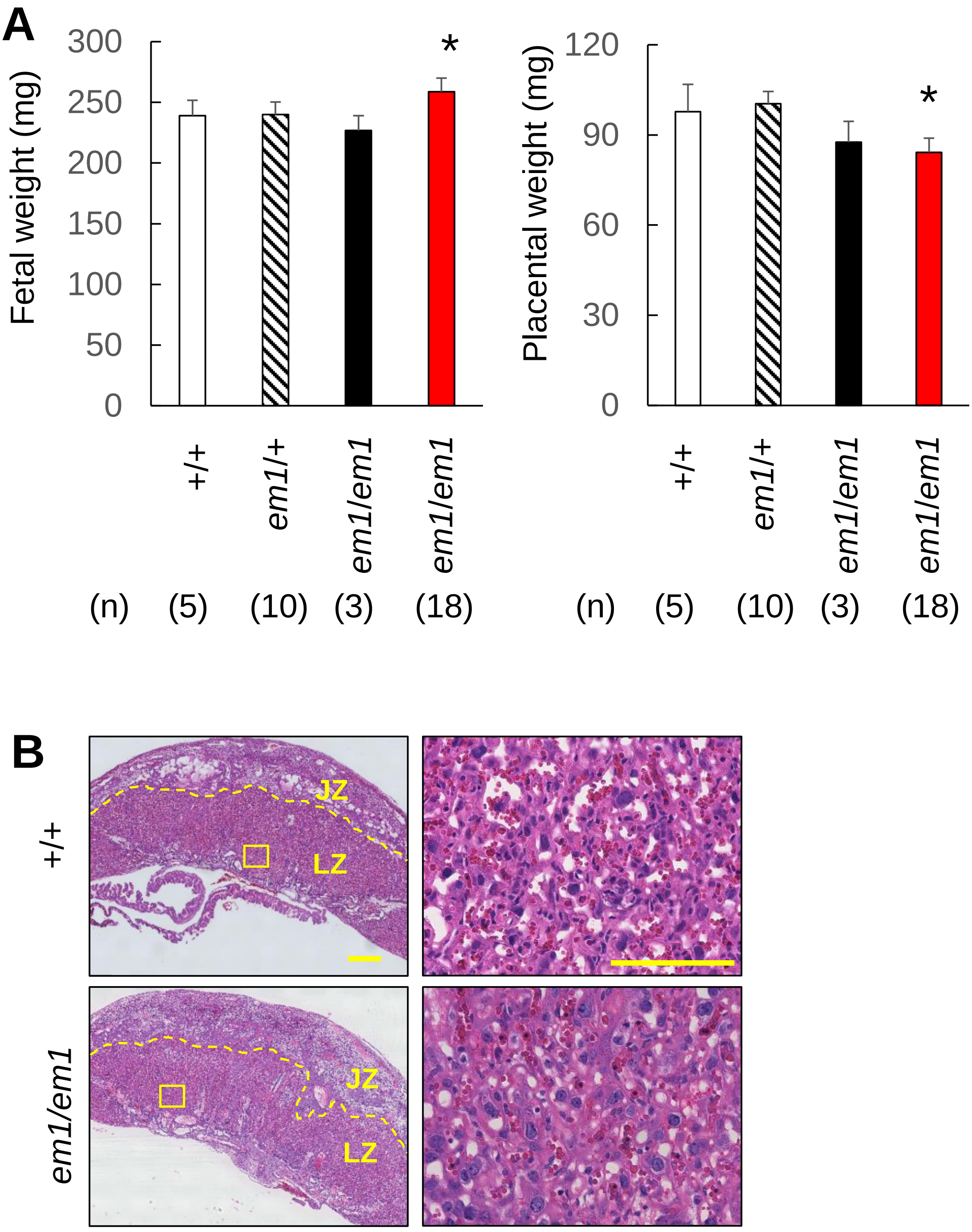

Supplementary Figure 1.

A. Fetal (Right) and placental (Left) weight recovered from E14.5 of heterozygous or homozygous matings. WT; wild-type, *: $Lvrn^{em1/em1}$ pups from $Lvrn^{em1/em1}$ male and female matings.

B. Left, hematoxylin-eosin staining of WT and homozygous placenta at E14.5. Dotted line demarcates the labyrinth zone (LZ) and junctional zone (JZ). Right, magnified images of boxed area in left. Scale Bars; 300 μm (Left), 100 μm (Right).
